# Inhibition of Biofilm Formation in *Cutibacterium acnes*, *Staphylococcus aureus*, and *Candida albicans* by the Phytopigment Shikonin

**DOI:** 10.3390/ijms25042426

**Published:** 2024-02-19

**Authors:** Yong-Guy Kim, Jin-Hyung Lee, Sang-Hun Kim, Sun-Young Park, Yu-Jeong Kim, Choong-Min Ryu, Hwi-Won Seo, Jin-Tae Lee

**Affiliations:** 1 School of Chemical Engineering, Yeungnam University, 280 Daehak-Ro, Gyeongsan 38541, Republic of Korea; yongguy7@ynu.ac.kr (Y.-G.K.); jinhlee@ynu.ac.kr (J.-H.L.); minimo017@ynu.ac.kr (S.-H.K.); sunyong3142@ynu.ac.kr (S.-Y.P.); 2 Biosystems & Bioengineering Program, University of Science and Technology (UST), Daejeon Campus, Daejeon 34113, Republic of Korea; yu9535@kribb.re.kr (Y.-J.K.); cmryu@kribb.re.kr (C.-M.R.); 3 Infectious Disease Research Center, Korea Research Institute of Bioscience and Biotechnology, Daejeon 34141, Republic of Korea

**Keywords:** *Candida albicans*, *Cutibacterium acnes*, polymicrobial biofilms, shikonin, *Staphylococcus aureus*

## Abstract

Skin microbiota, such as acne-related *Cutibacterium acnes*, *Staphylococcus aureus*, and fungal *Candida albicans*, can form polymicrobial biofilms with greater antimicrobial tolerance to traditional antimicrobial agents and host immune systems. In this study, the phytopigment shikonin was investigated against single-species and multispecies biofilms under aerobic and anaerobic conditions. Minimum inhibitory concentrations of shikonin were 10 µg/mL against *C. acnes*, *S. aureus*, and *C. albicans*, and at 1–5 µg/mL, shikonin efficiently inhibited single biofilm formation and multispecies biofilm development by these three microbes. Shikonin increased porphyrin production in *C. acnes*, inhibited cell aggregation and hyphal formation by *C. albicans*, decreased lipase production, and increased hydrophilicity in *S. aureus*. In addition, shikonin at 5 or 10 µg/mL repressed the transcription of various biofilm-related genes and virulence-related genes in *C. acnes* and downregulated the gene expression levels of the quorum-sensing *agrA* and *RNAIII*, α-hemolysin *hla*, and nuclease *nuc1* in *S. aureus*, supporting biofilm inhibition. In addition, shikonin prevented multispecies biofilm development on porcine skin, and the antimicrobial efficacy of shikonin was recapitulated in a mouse infection model, in which it promoted skin regeneration. The study shows that shikonin inhibits multispecies biofilm development by acne-related skin microbes and might be useful for controlling bacterial infections.

## 1. Introduction

Microbial biofilms are complex surface-attached communities of bacteria, fungi, and/or yeasts held together by a self-produced polymer matrix. Metabolically inactive biofilm cells encased in exopolymeric materials are protected from antimicrobial agents and play important roles in antimicrobial resistance and host immune system tolerance [1,2,3]. Polymicrobial biofilms are common in the environment and chronic infections, though most studies have addressed single-species biofilms. Hence, antibiofilm and antivirulence approaches are needed to control biofilm formation by single or several microbes [4,5,6].

Human skin is home to thousands of bacteria, fungi, and viruses. Like gut microbiota, skin microorganisms play essential protective roles against invading pathogens [7]. For example, *Propionibacterium* spp., *Staphylococcus* spp., *Corynebacterium* spp., and *Malassezia* spp. (a fungus) coexist in sebaceous follicles [7]. *Cutibacterium acnes* (formerly *Propionibacterium acnes*) forms biofilms with bacteria like *Staphylococcus aureus*, which are also involved in the pathogenesis of acne [8], and fungal *C. albicans* forms biofilms with *S. aureus*, *P. aeruginosa*, and *Enterococcus* species, which have greater antimicrobial/antifungal tolerance than corresponding single-species biofilms [9]. However, despite the interest shown in polymicrobial biofilms, little has been published on the control of multispecies biofilms.

**Figure 1:**
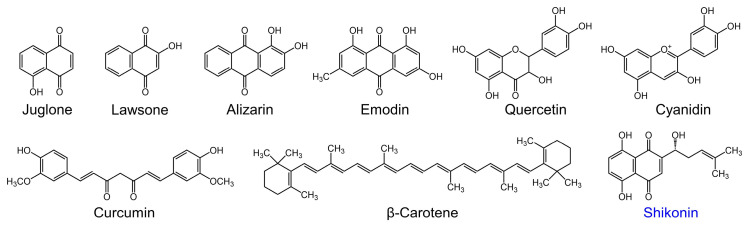
Structures of phytopigments with antimicrobial and antibiofilm activities.

Many phytochemicals have been reported to possess antioxidant, antimicrobial, and antibiofilm activities [10,11,12]. In particular, several phytopigments, such as juglone [13], lawsone [14], alizarin [15], emodin [16], quercetin [17], cyanidin [18], curcumin [19], and carotenoids [20], have been reported to have antimicrobial and antibiofilm activities (Figure 1).

In our ongoing effort to identify novel phytopigments with potent antimicrobial and antibiofilm activities, we focused on shikonin (Figure 1), based on its structural similarity to active phytopigments. Shikonin, sourced from the roots of *Lithospermum erythrorhizon* Siebold & Zucc, has been used as a traditional medicine in Asia and has no known harmful side effects [21]. Shikonin is a red pigment with well-established pharmacological properties, which include anticancer, antidiabetic, antiviral, anti-inflammatory, antioxidant, and antimicrobial effects [22,23,24]. Also, shikonin is used commercially as a pigment in the textile and cosmetics industries, as a food additive, and as a pH indicator in the food industry [25,26]. Notably, shikonin has antibacterial and antibiofilm effects on *S. aureus* [27,28], *C. albicans* [29,30,31], and *Listeria monocytogenes* [32], and hence, we hypothesized that shikonin possesses antibiofilm activities against acne-associated *C. acnes* and multispecies skin microbiota such as *C. acnes*, *S. aureus*, and *C. albicans. C. acnes* is a Gram-positive anaerobic bacterium implicated in acne vulgaris formation, whereas *S. aureus* is a Gram-positive facultative bacterium that causes skin infections, food poisoning, implant infections, and nosocomial infections [8]. On the other hand, *C. albicans* is a common fungal pathogen and the etiologic agent of candidiasis. Moreover, drug-resistant strains of these microbes are widespread due to the overuse of antibiotics and antifungal agents.

Hence, we investigated the antibiofilm activities of shikonin on single-species biofilms of *C. acnes*, *S. aureus*, and *C. albicans* under aerobic and anaerobic conditions and three-species mixed biofilms under anaerobic conditions. This is the first report of the antibiofilm activities of shikonin against *C. acnes* and multiple species. To investigate how shikonin inhibits biofilm formation, confocal laser scanning microscopy (CLSM) and scanning electron microscopy (SEM) were used to investigate morphological changes, biofilm formation, cell aggregation, and the hyphal growth of *C. albicans.* In addition, cell surface hydrophilicity, cell agglutination, porphyrin production, and lipase production were examined, and transcriptomic analysis was performed. Also, an ex vivo porcine skin model and in vivo mouse model were used to confirm the antibiofilm effects of shikonin, and *Caenorhabditis elegans* and *Brassica rapa* growth models were used to investigate its toxic and ecological effects.

## 2. Results

### 2.1. Antimicrobial and Antibiofilm Activities of Shikonin against C. acnes, S. aureus, and C. albicans

**Figure 2:**
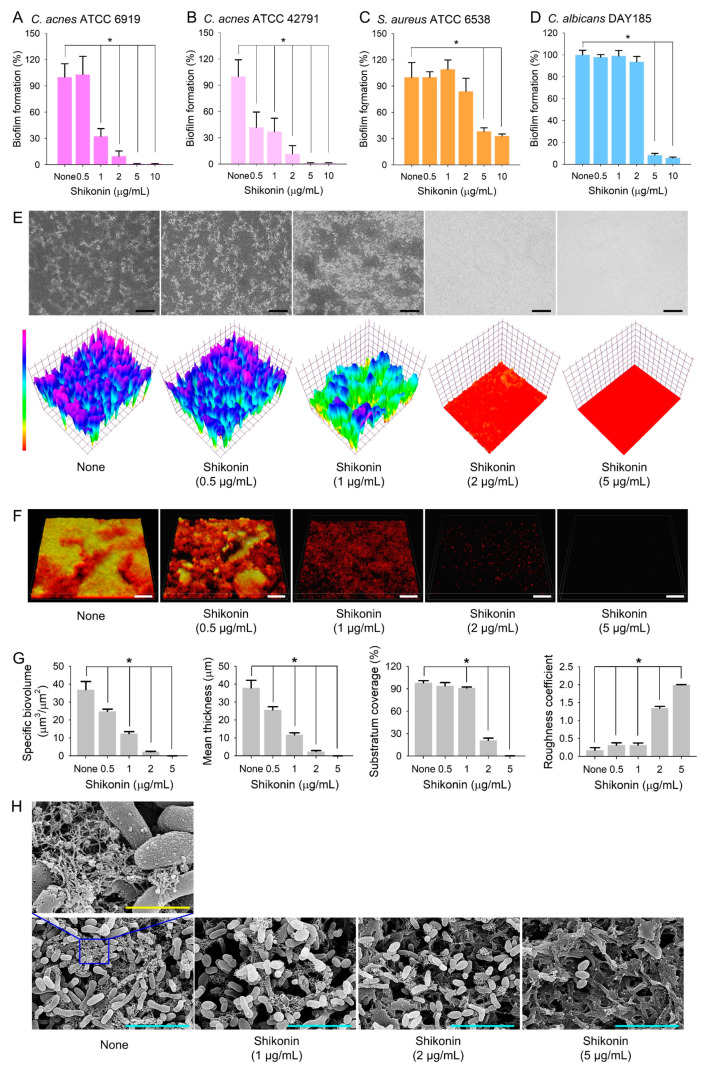
Antibiofilm activity of shikonin. (**A**–**D**) Biofilm formation by *C. acnes*, *S. aureus*, and *C. albicans* in the presence of shikonin under anaerobic conditions; (**E**) color-coded images of *C. acnes* biofilms; (**F**) CLSM images of *C. acnes* biofilms; (**G**) COMSTAT results; and (**H**) SEM images. Black and white bars represent 100 µm, and yellow and cyan bars represent 1 and 5 µm, respectively. None: non-treated control. *, *p* < 0.05 vs. non-treated controls (None).

The antimicrobial activity of shikonin was also assessed using an MIC assay and a planktonic cell growth curve. Shikonin had an MIC of 10 µg/mL after 7 days of culture, and planktonic growth curves showed that shikonin at 5 or 10 µg/mL significantly delayed *C. acnes* cell growth but only had marginal effects at concentrations up to 2 µg/mL (Figure S1A). And the antibiofilm activity of shikonin was analyzed against two *C. acnes* strains, ATCC 6919 and KCCM 42791, cultured in 96-well polystyrene plates under anaerobic conditions for 7 days. Shikonin dose-dependently inhibited biofilm formation by the two *C. acnes* strains (Figure 2A,B); for example, shikonin at 2 and 5 µg/mL inhibited *C. acnes* biofilm formation by 90% and 98%, respectively. Biofilm inhibitions were similar for the two *C. acnes* strains, and thus, the ATCC 6919 strain was used in further experiments.

The antimicrobial and antibiofilm activities of shikonin were also investigated against *S. aureus* and *C. albicans* under aerobic and anaerobic conditions. Shikonin had an MIC of 10 µg/mL against both microbes, and shikonin at concentrations >5 µg/mL significantly delayed the cell growth of *S. aureus* (Figure S1B,C). Furthermore, biofilm formation by *S. aureus* and *C. albicans* was dose-dependently inhibited by shikonin after culture for 1 day under aerobic conditions (Figure S1D, E). Notably, shikonin at 10 µg/mL abolished biofilm formation by *S. aureus* and *C. albicans* (Figure S1D,E), which was attributed to growth inhibition. These observations confirmed previous results for the antibacterial activity of shikonin against *S. aureus* (MIC 8–15 µg/mL) [22] and its antibiofilm activity against *C. albicans* [24,26]. Also, under anaerobic conditions, shikonin at 5 µg/mL inhibited *S. aureus* biofilm formation by 70% (Figure 2C) and inhibited *C. albicans* biofilm formation by 90% (Figure 2D). Additionally, shikonin dose-dependently inhibited two-component biofilm formation by *S. aureus* and *C. albicans* (*S. aureus*/*C. albicans*) in a 1:1 mixture of LB and PDB (Figure S1F). Since the antibiofilm effects of shikonin were previously reported for single-component *S. aureus* or *C. albicans* biofilms, we focused on polymicrobial biofilms with *C. acnes*.

*C. acnes* biofilm inhibition by shikonin was investigated by bright-field microscopy, CLSM microscopy, and SEM. Two- and three-dimensional biofilm images showed that shikonin at 1–5 µg/mL significantly inhibited biofilm formation on polystyrene plates (Figure 2E). CLSM showed shikonin dose-dependently inhibited *C. acnes* biofilm formation and that untreated controls produced dense biofilms (Figure 2F). Biofilm inhibition was quantified by biofilm quantification software COMSTAT analysis, which showed that shikonin at 2 and 5 µg/mL dramatically reduced biofilm mass, average thickness, and substratum coverage and increased the roughness coefficient (Figure 2G). Furthermore, SEM analysis showed shikonin reduced the number of attached cells on nylon membranes and, more importantly, the production of EPS (Figure 2H). Since EPS production is a hallmark of biofilm formation, it would appear that shikonin reduced biofilm formation by inhibiting EPS production in *C. acnes*.

### 2.2. Effects of Shikonin on Biofilm- and Virulence-Related Factors

**Figure 3:**
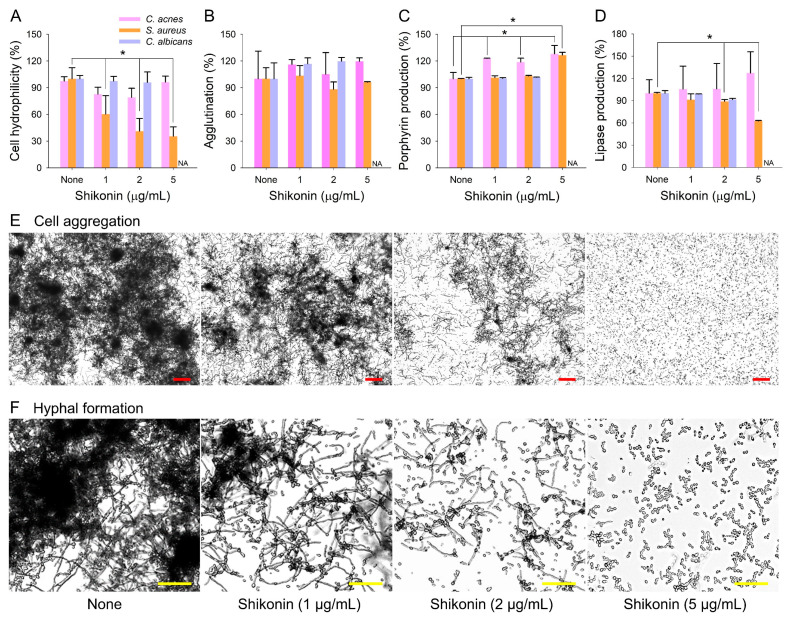
Inhibition of virulence factor production by shikonin. (**A**) Cell surface hydrophilicity, (**B**) cell agglutination, (**C**) porphyrin production, (**D**) extracellular lipase production, (**E**) cell aggregation, and (**F**) hyphal formation by *C. albicans*. Error bars indicate standard deviations. N/A means not applicable because of no cell growth under anaerobic conditions. *, *p* < 0.05 vs. non-treated controls (None). Red and yellow bars represent 100 and 20 µm, respectively.

To examine how shikonin affects biofilm formation, the cell surface hydrophilicity, cell agglutination, porphyrin production, and lipase production of *C. acnes*, *S. aureus*, or *C. albicans* were investigated. Shikonin at concentrations ≤2 µg/mL did not affect the cell surface hydrophilicity of *C. acnes* or *C. albicans* and dose-dependently decreased that of *S. aureus* (Figure 3A). Cell agglutination was unaffected by shikonin (Figure 3B). It has been reported that porphyrin reduces biofilm formation by *S. aureus* [33] and *Pseudomonas aeruginosa* [34]. Here, we observed shikonin increased porphyrin production by *C. acnes* (Figure 3C). In addition, shikonin decreased the activity of extracellular lipases in *S. aureus* but not in *C. acnes* or *C. albicans* (Figure 3D). In *C. albicans*, hyphal formation and cell aggregation are prerequisites for biofilm formation, and shikonin markedly inhibited both (Figure 3E,F) and at 5 µg/mL prevented aggregation and hyphal transition. A recent report demonstrated that shikonin inhibited the biofilm formation and hyphae transition of *C. albicans* via the RasI-cAMP-EfgI signaling pathway [31]. Also, molecular modeling studies reported that the fungicidal activity of shikonin was likely related to ergosterol complexation [30]. The current result supports the previous findings of the action mechanism.

### 2.3. Shikonin Inhibited Polymicrobial Biofilm Formation on Abiotic and Biotic Surfaces

**Figure 4:**
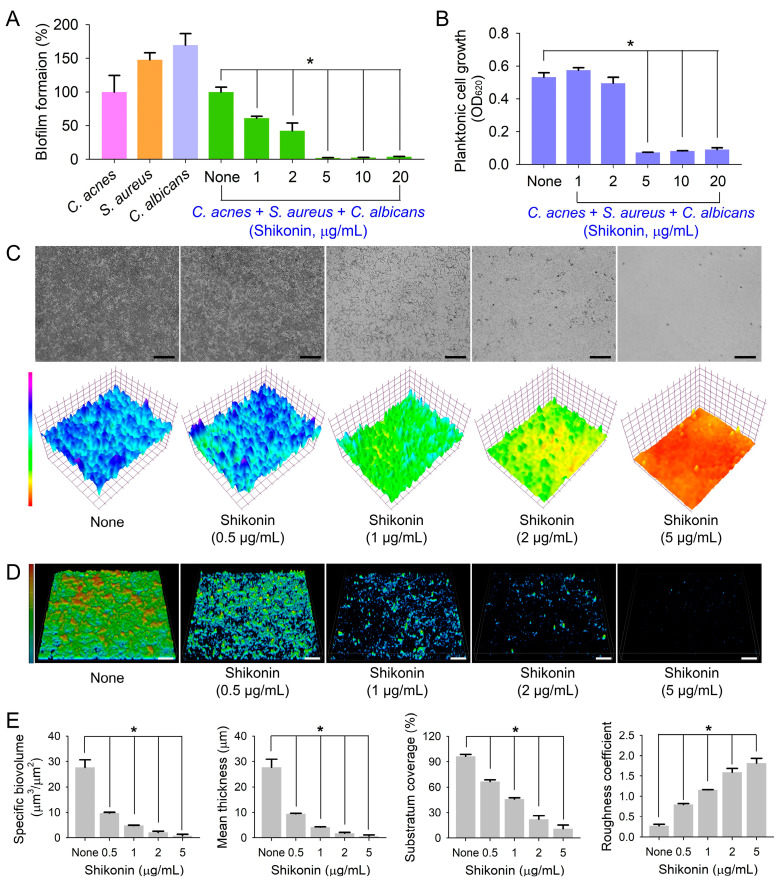
Inhibitory effects of shikonin on multispecies biofilms. (**A**) The antibiofilm effects of shikonin on *C. acnes*/*S. aureus*/*C. albicans* biofilms after 7-day culture under anaerobic conditions, (**B**) planktonic cell growth of multiple species, (**C**) color-coded images of three-species biofilms, (**D**) CLSM images of the three-component biofilms, and (**E**) COMSTAT results. Black and white bars indicate 100 µm. None: non-treated control. *, *p* < 0.05 vs. non-treated controls (None).

Recently, a polymicrobial biofilm model of *C. acnes*, *S. aureus*, and *C. albicans* was developed using a mixed RCM, LB, and PDB (1:1:1) medium and anaerobic conditions [35]. Hence, we investigated the antibiofilm activity of shikonin on this three-species model in polystyrene plates. In a mixed medium, all three microbes effectively formed biofilms, and in combination, the three microbes produced multispecies biofilms (Figure 4A). Shikonin dose-dependently inhibited polymicrobial biofilm formation (Figure 4A); for example, at 2 and 5 µg/mL, shikonin inhibited biofilm development by 57% and 98%, respectively, after culture for 7 days. It appears that the multispecies biofilm reduction by shikonin at 5 µg/mL is partially due to the inhibition of cell growth, but shikonin at 2 µg/mL inhibited biofilm formation without affecting planktonic cell growth (Figure 4B).

**Figure 5:**
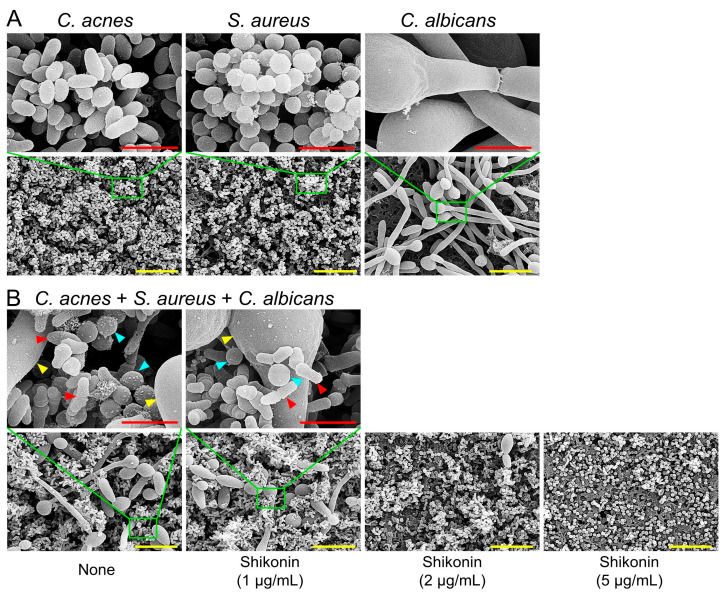
Microscopic observations of multispecies biofilms. (**A**) SEM images of single-species biofilms and (**B**) a three-component biofilm formed in the presence of shikonin after culture under anaerobic conditions for 7 days. Red and yellow scale bars represent 3 and 10 µm, respectively. Red, cyan, and yellow triangles indicate cells of *C. acnes*, *S. aureus*, and *C. albicans*, respectively. None: non-treated control.

Microscopic studies confirmed the inhibitory effect of shikonin on multispecies biofilm production. Blue 3D color images were obtained for non-treated biofilms, indicating abundant biofilm formation, whereas green to red images were obtained at shikonin concentrations of 1–5 µg/mL, indicating poor or no biofilm formation (Figure 4C). Also, CLSM and COMSTAT analysis confirmed this inhibitory effect (Figure 4D,E). In the absence of shikonin, the three microbes formed substantial multispecies biofilms (~25 µm thick with 100% surface coverage), while in the presence of shikonin at 1–5 µg/mL, biofilm densities and thicknesses were dramatically decreased (Figure 4E). SEM was used to investigate microbial morphologies (Figure 5A,B). Non-treated *C. acnes* had rod-type cells, *S. aureus* had round cells, and *C. albicans* produced large hyphae and yeast cells. In mixed *C. acnes*/*S. aureus*/*C. albicans* biofilms, the three microbes were admixed. Shikonin at 2 µg/mL reduced the number of *C. albicans* cells, and at 5 µg/mL, only *S. aureus* cells remained.

### 2.4. Shikonin Altered Gene Expression Levels in C. acnes and S. aureus

**Figure 6:**
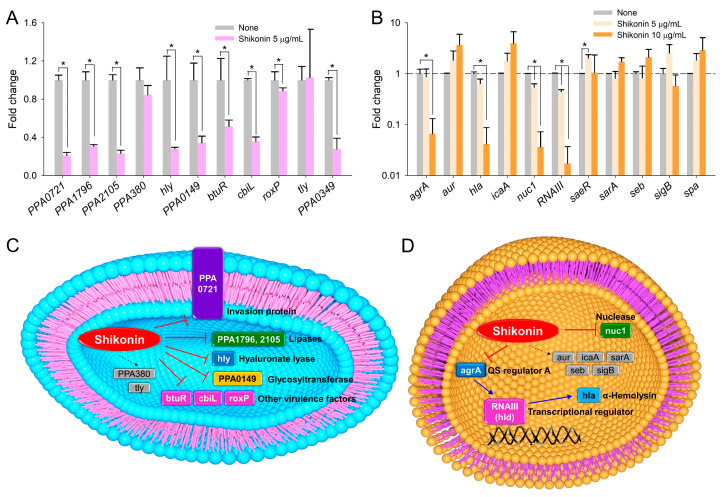
Effects of shikonin on gene expression and putative mechanisms. Relative transcriptional profiles of biofilm-related genes in (**A**) *C. acnes* and (**B**) *S. aureus*. *C. acnes* cells were treated with shikonin at 5 μg/mL for 24 h without shaking under anaerobic conditions, and *S. aureus* cells were treated with shikonin at 5 and 10 μg/mL for 3 h with 250 rpm shaking under aerobic conditions. Fold changes indicate transcriptional differences observed in treated vs. untreated (None) cells as determined by qRT-PCR. *16s rRNA* was used as the housekeeping gene for *C. acnes* and *S. aureus*. *, *p* < 0.05 vs. non-treated controls. Diagram of the putative mechanisms of shikonin in (**C**) *C. acnes* and (**D**) *S. aureus*. Blue arrows (→) indicate upregulation of gene expression or positively affecting a phenotype, red arrows (˫) indicate downregulation of gene expression or negatively affecting a phenotype, and black dotted arrows indicate no change/no effect.

To understand the molecular mechanism of shikonin in *C. acnes*, the differential expression of 11 biofilm- and virulence-related genes was examined by real-time qRT-PCR. Shikonin was found to modulate the transcriptional expression levels of several lipase genes, hyaluronate lyase genes, and virulence-related genes (Figure 6A) but not to alter the expression of the housekeeping gene (*16s rRNA*). Notably, the expression levels of adhesin/invasion (PPA0721), lipase (PPA1796 and PPA2105), hyaluronate lyase (*hly*), and virulence-related genes (PPA0149, *btuR*, *cbiL*, *roxP*, and PPA0349) were downregulated by shikonin at 5 µg/mL, while the expression levels of hyaluronate lyase (PPA380) and hemolysin (*tly*) were not affected.

Also, the expression levels of 11 biofilm- and virulence-related genes were examined by real-time qRT-PCR to investigate the mechanisms underlying the antibiofilm effects of shikonin against *S. aureus*. Shikonin at 10 µg/mL downregulated the expression levels of quorum-sensing regulator *agrA*, α-hemolysin *hla*, nuclease *nuc1*, and quorum-sensing-regulatory *RNAIII* more than 10-fold, whereas the expression levels of the other six genes tested were relatively unaffected (Figure 6B). In particular, shikonin downregulated the expression levels of *agrA* and *RNAIII* 15- and 58-fold, respectively.

### 2.5. Shikonin Prevented the Attachment of C. acnes and Other Microbes to Porcine Skin

Since *C. acnes*, *S. aureus*, and *C. albicans* are common inhabitants of animal skin, we investigated the ability of shikonin to prevent them from forming biofilms on porcine skin. SEM analysis showed that shikonin at 1 and 5 µg/mL markedly reduced *C. acnes* attachment (Figure S2A), and at the same concentrations, it prevented biofilm formation by *C. acnes*/*S. aureus*/*C. albicans* (Figure S2B).

### 2.6. Antimicrobial and Wound-Healing Efficacy of Shikonin against Polymicrobial Infection

**Figure 7:**
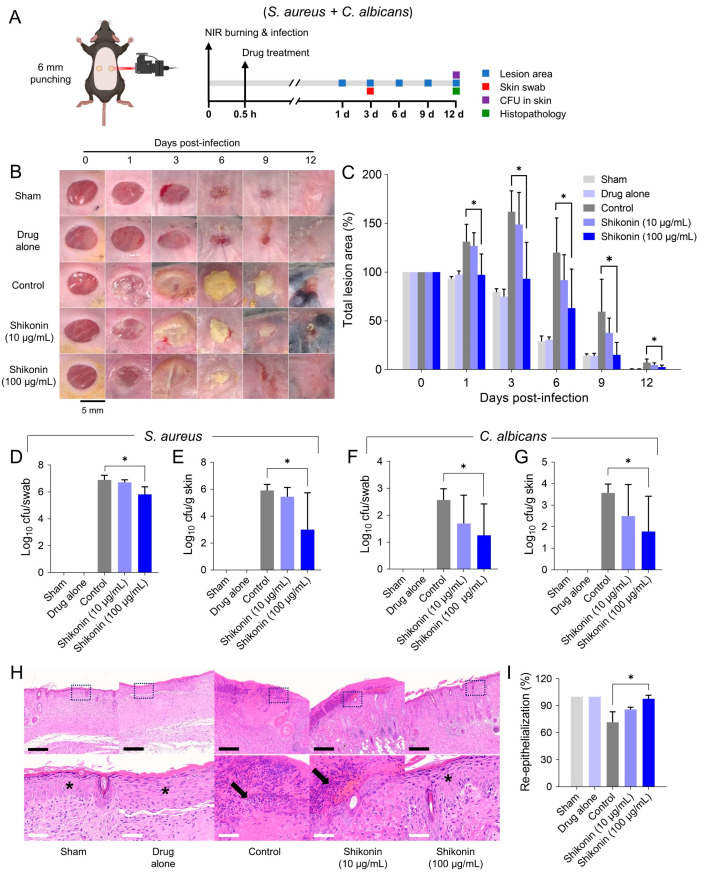
Efficacy of shikonin in mice. Antimicrobial and skin wound-healing effects of shikonin on combined *S. aureus* and *C. albicans* infection. (**A**) Schematic of the experimental protocol used for the skin wound/infection mouse model. NIR: near-infrared. (**B**) Skin lesion areas were monitored from DPI 0 to 12 and are presented as (**C**) the percentages of total lesion areas to initial skin incision areas. (**D**,**F**) Bacterial counts of skin swabs at DPI 3 and (**E**,**G**) tissues at DPI 12 for *S. aureus* and *C. albicans*, respectively. (**H**) Hematoxylin and eosin-stained tissues were prepared for histopathological evaluation, and (**I**) re-epithelialization rates were calculated by measuring mean epithelial regeneration areas. Photomicrographs of epithelial layers (upper images; (**H**)) and corresponding higher magnification images (lower images; (**H**)) (original magnifications 100 and 400×, respectively) showing patterns of skin remodeling. Drug-alone means non-infected mice treated with 100 μg/mL of shikonin. Asterisks indicate regenerated epithelium, and arrows indicate necrotic tissue and inflammatory cells. (**H**) Black scale bars represent 200 µm and white scale bars 50 µm. Means ± standard deviations were calculated for 3 to 5 mice. *, *p* < 0.05 vs. infected non-treated controls.

A mouse model of skin wound/infection was used to evaluate the antimicrobial and wound-healing efficacies of shikonin against combined *S. aureus* and *C. albicans* infection (Figure 7A). Initially, we optimized the amounts of infection and burning required to induce skin infections (Figure S3A–C). A single treatment with shikonin at 100 µg/mL (50 µg/kg mice) significantly reduced lesion area by 78% between DPI 1 and DPI 12 as compared with the sham group and completely closed wounds at DPI 12, whereas at 10 µg/mL, it had minor effects (Figure 7B,C). In addition, shikonin at 100 µg/mL reduced areas of yellowish purulent discharge and caused their early detachment at DPI 6 and 9 (Figure 7B). We included the drug-alone group to evaluate the skin toxicity of shikonin. In this group, shikonin at 100 µg/mL exhibited the same skin restoration pattern as the sham group (Figure 7B,C). Furthermore, shikonin treatment at 100 µg/mL reduced the bacterial counts of *S. aureus* and *C. albicans* in skin swabs by ~1 and 2 log_10_ cfu, respectively, at DPI 3 (Figure 7D,F). In addition, shikonin treatment at 100 µg/mL reduced the bacterial burdens of *S. aureus* and *C. albicans* in skin homogenates by ~3 and 2 log_10_ cfu, respectively, at DPI 12 (Figure 7E,G).

Histopathological evaluation on DPI 12 showed that re-epithelialization was >95% in the shikonin 100 µg/mL group and ~70% in the control group (Figure 7I). In addition, less purulent necrotic tissue with inflammatory cells was observed in the shikonin 100 µg/mL group than in the control group (Figure 7H,I). Furthermore, animals in the sham and drug-alone groups showed 100% re-epithelialization and no inflammatory lesions, indicating that shikonin had no skin toxicity. Moreover, spleen histopathology showed a higher white pulp ratio in the shikonin 100 µg/mL group than in the control group (Figure S3D), indicating that shikonin did not cause secondary systemic lesions.

### 2.7. Toxicity of Shikonin in a Plant Model and a Nematode Model

**Figure 8:**
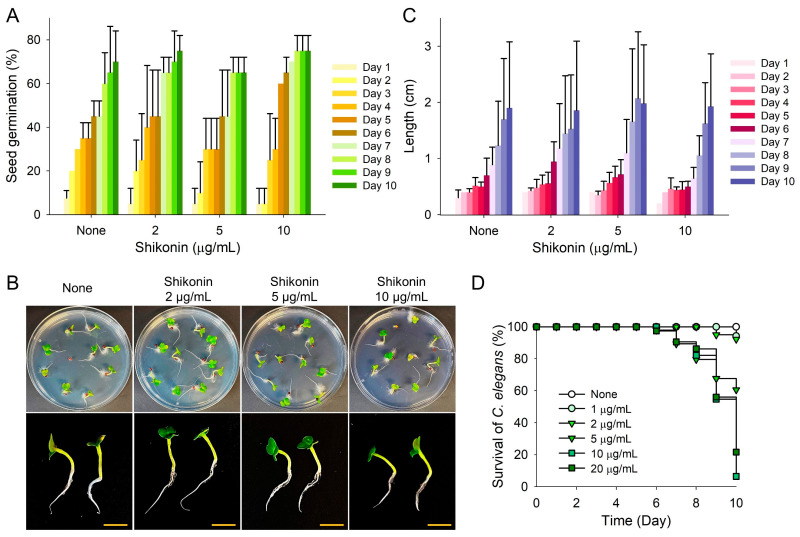
Toxicities of shikonin in the plant germination and nematode models. (**A**) *B. rapa* seed germination was performed using Murashige and Skoog agar medium supplemented with or without shikonin at 25 °C. (**B**,**C**) Plant total lengths were analyzed over 10 days. Yellow scale bars indicate 1 cm. (**D**) *C. elegans* survival was assessed in the presence and absence of shikonin for 10 days.

The pharmacological properties of shikonin have been widely studied [36], and thus, we performed toxicity assessments using a plant (*Brassica rapa*) growth model and a nematode (*Caenorhabditis elegans*) model. The germination rate of *B. rapa* was unaffected by shikonin at 2, 5, or 10 µg/mL (Figure 8A), and growth and height were not affected for 10 days after shikonin treatment (Figure 8B,C). Hence, the phytopigment shikonin exerts antibiofilm activity against three microbes without affecting plant growth. In the *C. elegans* model, shikonin at concentrations of ≤20 µg/mL had no toxic effect after culture for 7 days, whereas culture for longer periods exhibited dose-dependent toxicity (Figure 8D). These results indicate that shikonin at its active antibiofilm concentration range (1–10 µg/mL) was not toxic to *B. rapa* growth and was mildly toxic to *C. elegans*.

## 3. Discussion

Multispecies biofilms are common in nature and often aggravate bacterial and fungal infections. This study shows that phytochemical shikonin at 2–5 µg/mL is an effective antibiofilm agent against polymicrobial biofilms formed by *C. acnes*, *S. aureus*, and/or *C. albicans* in vitro, ex vivo, and in vivo and provides clues regarding the mechanisms involved.

Plant pigments, such as anthocyanins, carotenoids, and betalains, are widely used to attract insects, birds, and animals and protect plants from UV light [37]. Furthermore, many phytopigments possess antioxidative and antimicrobial activities [18]. In this study, we propose that shikonin exerts antibiofilm activity against several microbes to protect plants from microbial attachment and biofilm formation, which is in line with the findings of recent studies on the antibiofilm activities of phytopigments (Figure 1).

Shikonin contains a 1,4-naphthoquinone motif and three additional hydroxyl and methyl groups, and thus, it exhibits similarities with antibiofilm pigments such as juglon, lawsone, alizarin, and emodin (Figure 1). Moreover, it has been reported that the antibiofilm activities of flavonoids [17] and anthraquinones [15] against *S. aureus* and *C. albicans* are related to the number and positions of hydroxyl groups. The current study supports the importance of hydroxyl groups for antibiofilm activity.

Although the antibiofilm activities of shikonin against several bacteria, such as *Pseudomonas aeruginosa* [38], *Listeria monocytogenes* [32], *S. aureus* [28], and *C. albicans* [29,30,31], have been reported, the mechanism involved has not been fully elucidated. In addition, it has been suggested that shikonin acts as a quorum-sensing inhibitor against *P. aeruginosa* [38] and downregulates the expression levels of several hyphae- and biofilm-related genes in *C. albicans* [29]. Our results (Figure 3 and Figure 5) support the notion that hyphal growth inhibition plays a major role in biofilm inhibition in *C. albicans*, which confirms previous important findings [30,31]. Obviously, the mechanism of shikonin differs in the other two tested microbes.

In *C. acnes*, shikonin significantly downregulated the expression of several adhesin, lipase, hyaluronate lyase, and virulence-related genes (Figure 6A). Previously, it was reported that *C. acnes* biofilm cells produce more extracellular lipases than planktonic cells [35,39], which is supported by our observation that shikonin reduced the expression levels of adhesin (PPA0721) and lipase genes (PPA1796, and PPA2105). Furthermore, *C. acnes* is known to possess a hyaluronate lyase gene (*hly*), the protein product of which degrades hyaluronic acid, an extracellular matrix component of the epidermis [40]. Interestingly, shikonin repressed the gene expression levels of *hly* and those of the antioxidant and acne pathogenesis-related genes *btuR* and *cbiL*, which suggests shikonin might inhibit host tissue invasion (Figure 6C). 

In *S. aureus*, shikonin significantly downregulated the expression levels of quorum-sensing regulator *agrA*, quorum-sensing-regulatory *RNAIII*, α-hemolysin *hla*, and nuclease *nuc1* (Figure 6B). These findings are consistent with previous research indicating the crucial role of these factors in *S. aureus* pathogenicity and biofilm formation. The regulatory RNA molecule RNAIII binds to quorum-sensing accessory gene regulator A (AgrA) and positively regulates various virulence factors and biofilm development [41], and α-hemolysin and lipase play positive roles in *S. aureus* biofilm formation [42,43]. Also, the structurally similar pigments alizarin and quercetin inhibited the hemolytic activity of *S. aureus* and its ability to form biofilms [15,17], further highlighting the potential of natural compounds in combating bacterial virulence. A recent report demonstrated that shikonin at sub-inhibitory concentrations repressed the gene expression of *hla*, staphylococcal enterotoxin A (*sea*), and staphylococcal enterotoxin B (*seb*) [28]. The current results support the previous findings. Also, shikonin along with other quinone derivatives are potent inhibitors of another target for biofilm inhibition, the sortase [44]. Taking these results together, it appears that shikonin might inhibit bacterial quorum sensing, virulence factor production, and biofilm formation (Figure 6D). Collectively, our findings suggest that shikonin may exert its antibacterial effects through multiple mechanisms, including inhibition of bacterial quorum sensing, suppression of virulence factor production, and interference with biofilm formation. These insights underscore the potential of shikonin as a promising therapeutic agent for combating *S. aureus* infections, warranting further investigation into its mechanisms of action and therapeutic applications.

The wound infection model used in the present study is a gold standard for preclinical evaluations of antimicrobial agents targeting skin and soft tissue infections [45]. An *S. aureus*/*C. albicans* polymicrobial infection model was established (Figure S3A–C), as it was demonstrated in a previous study that *S. aureus* and *C. albicans* synergistically enhance virulence and antimicrobial resistance [46]. Since a one-time treatment with shikonin (50 µg/kg mice) showed significant antimicrobial efficacy in the mouse infection model without toxicity, multiple administrations and higher doses are possible. Although mild hyperplasia in the basal layer was observed during epithelialization, accelerated wound closure by shikonin could prevent the establishment of infections by other pathogens. In addition, the anti-inflammatory effect of shikonin induced by inhibitions of TNF-α and NO production reported in previous studies might facilitate skin regeneration and its antimicrobial activity [47]. Thus, we believe a study of the regulations of microbial and host factors by shikonin would increase understanding of the mechanism responsible for its skin-healing and antimicrobial effects.

Shikonin has many properties of pharmacological interest coupled with low cytotoxicity [22]. Furthermore, a recent pharmacokinetic study concluded that shikonin does not violate Lipinski’s rule and exhibits drug-likeness properties for oral bioavailability [48]. Our toxicity results confirm that shikonin is non-toxic to plants and mice and mildly toxic to nematodes (Figure 8). These results further support the viability of shikonin as a candidate for pharmaceutical development, warranting further exploration of its therapeutic applications and safety profiles.

Since several phytopigments have been reported to have antimicrobial activities, the current study demonstrated that the phytopigment shikonin exhibited potent antimicrobial and antibiofilm activities. Shikonin at a few µg/mL efficiently inhibited single-species and multispecies biofilm formation by *C. acnes*, *S. aureus*, and fungal *C. albicans*. Transcriptional analyses and phenotypic assays partially revealed its mode of action in these microbes. Furthermore, the ex vivo porcine skin and in vivo mouse model studies confirmed the antibiofilm efficacy of shikonin, and the nematode and plant growth models demonstrated its relatively non-toxic nature.

## 4. Materials and Methods

### 4.1. Strains, Chemicals, and Culture Materials

*C. acnes* KCCM 41747 (ATCC 6919 derived from human facial acne), *C. acnes* KCCM 42791 (derived from dental plaque), methicillin-sensitive *S. aureus* ATCC 6538, and fluconazole-resistant *C. albicans* strain DAY185 were used in this study. All microorganisms were obtained from the American Type Culture Collection (ATCC) or the Korean Culture Center of Microorganisms (KCCM). *C. acnes* was cultured on Reinforced Clostridium Media (RCM) agar plates for colony preparation and in liquid RCM at 37 °C under anaerobic conditions, and BD GasPak™ EZ Gas Generating Anaerobic Pouch Systems (Fisher Scientific, Pittsburgh, PA, USA) were used for all other experiments. *S. aureus* was cultured in LB (Luria-Berani) medium at 37 °C, and the *C. albicans* strain was cultured in potato dextrose broth (PDB) medium at 37 °C. Shikonin was purchased from Sigma-Aldrich (St. Louis, MO, USA). Dimethyl sulfoxide (DMSO) served as the solvent for shikonin dissolution, while a concentration of 0.1% (*v*/*v*) DMSO acted as the negative control. At this specific concentration, there was no discernible impact on bacterial growth or biofilm formation.

### 4.2. Planktonic Cell Growth Measurements and MIC Determinations

To investigate the effect of shikonin on the planktonic cell growths of *C. acnes*, *S. aureus*, and *C. albicans*, 7-day cultures of *C. acnes* or overnight cultures of *S. aureus* and *C. albicans* were re-inoculated (dilution 1:50 for *C. acnes* and *C. albicans* and 1:100 for *S. aureus*) in RCM, LB, and PDB media, respectively. The cultures were treated with different concentrations of shikonin (0, 1, 2, 5, or 10 µg/mL) in 96-well plates. Plates were incubated for 7 days at 37 °C under anaerobic conditions using anaerobic pouches (Fisher Scientific, Pittsburgh, PA, USA), and cell turbidities were then measured at 620 nm using a Multiskan EX microplate reader (Thermo Fisher Scientific, Waltham, MA, USA).

The determination of minimum inhibitory concentrations (MICs) followed a modified protocol inspired by the Clinical Laboratory Standards Institute (CLSI) guidelines for both bacteria and yeasts [14]. The MIC was characterized as the minimal concentration capable of halting cell growth. The experiments were conducted across a minimum of three separate culture instances.

### 4.3. Biofilm Assays in 96-Well Plates

Biofilms were produced on 96-well polystyrene plates, as previously described [35]. Briefly, overnight cultures at an initial turbidity of OD_600_ 0.05 (~10^8^ or 10^7^ CFU/mL) for *S. aureus* and *C. acnes* and OD_600_ 0.1 (~10^5^ CFU/mL) for *C. albicans* were inoculated into appropriate culture media (final volume 300 μL) with or without shikonin (0, 0.5, 1, 2, 5, or 10 μg/mL) and incubated for 24 h under aerobic conditions for *S. aureus* and *C. albicans* or 7 days under anaerobic conditions for the mixed cultures of *C. acnes*, *S. aureus*, and *C. albicans* at 37 °C without shaking. After planktonic cells were eliminated through three washes with sterile water, the biofilm cells adhered to 96-well plates (SPL Life Sciences, Pocheon, Republic of Korea) were subjected to staining with 0.1% crystal violet (Sigma-Aldrich, St. Louis, MO, USA) for 20 min at room temperature. Subsequently, they were rinsed repeatedly with sterile distilled water and suspended in 95% ethanol. Measurements were taken at 570 nm using a plate reader, and the outcomes represent the averages derived from a minimum of six repetitions across three independent cultures. Inhibition ratios were calculated by expressing the absorbance readings of treated biofilms as percentages of untreated controls.

### 4.4. Biofilm Observations by Live Imaging Microscopy, CLSM, and SEM

To observe *C. acnes*, *S. aureus*, and *C. albicans* biofilms or mixed *C. acnes*/*S. aureus*/*C. albicans* biofilms grown in the presence or absence of shikonin (0, 0.5, 1, 2, 5, or 10 μg/mL), cells (300 μL) were incubated at 37 °C under anaerobic conditions using anaerobic pouches for 7 days [35]. Following the incubation period, planktonic cells were eliminated through three washes with sterile water, and biofilms were visualized using live imaging microscopy using the iRiS^TM^ Digital Cell Imaging System (Logos BioSystems, Anyang, Republic of Korea). Biofilm images were reconstructed into color-coded 2D and 3D representations using ImageJ (https://imagej.nih.gov/ij/index.html (accessed on 12 September 2022)).

For CLSM, single- and three-species biofilms were formed on 96-well polystyrene plates in the presence or absence of shikonin without shaking for 7 days under anaerobic conditions at 37 °C. Planktonic cells were then removed by washing with distilled water three times, and biofilms were stained with carboxyfluorescein diacetate succinimidyl ester (Invitrogen, Molecular Probes, Inc, Eugene, OR, USA). Plate bases were then visualized using a 488 nm Ar laser (emission 500 to 550 nm) under a confocal laser microscope (Nikon Eclipse Ti, Tokyo, Japan). Two independent cultures were performed under each experimental condition, and at least 12 random positions were assayed for COMSTAT analysis [41] to quantify the biofilm formation. 

SEM was utilized to examine the formation of single-species or multispecies biofilms on nylon membranes, following established procedures [49]. Briefly, a nylon membrane (0.2 µm pore size, Merck Millipore, Burlington, MA, USA) was cut into 0.4 × 0.4 cm pieces. These pieces were then positioned in 96-well plates containing single or mixed species cultures, cultivated in the presence or absence of shikonin for 7 days under anaerobic conditions at 37 °C. RCM medium was used to produce *C. acnes* biofilms. To produce *C. acnes*/*S. aureus*/*C. albicans* biofilms, a 1:1:1 mixture of LB, RCM, and PDB media was used. Cells that adhered to the nylon membrane were fixed with a solution containing glutaraldehyde (2.5%) and formaldehyde (2%) for 24 h, post-fixed using osmium tetroxide, and dehydrated through a 50, 70, 80, 90, 95, 100% ethanol series and isoamyl acetate. After critical-point drying, biofilm cells were examined and imaged using an S-4800 scanning electron microscope (Hitachi, Tokyo, Japan) at a voltage of 15kV.

### 4.5. C. albicans—Hyphal Formation and Cell Aggregation Assay

A microscopic live cell imaging system was used to observe filamentous growth and hyphal formation by *C. albicans*, as previously described [49]. Briefly, *C. albicans* cells were re-inoculated at 1:50 dilution in 2 mL of PDB medium containing shikonin (0, 1, 2, or 5 μg/mL) in 14 mL tubes and incubated for 24 h at 37 °C without shaking. Cells were subsequently observed utilizing an iRiS^TM^ Digital Cell Imaging System (Logos BioSystems, Anyang, Republic of Korea); at least four independent cultures were used. To assess cell aggregation, *C. albicans* cells were inoculated at a dilution of 1:50 into 1 mL of PDB in 1.7 mL tubes and incubated at 37 °C without shaking for 24 h. Following incubation, cells were visualized under a bright field using the iRiS^TM^ Digital Cell Imaging System. At least four independent experiments were performed.

### 4.6. Cell Surface Hydrophilicity Assay

Cell surface hydrophilicity was assessed as previously reported [50]. Briefly, after incubating cells of *C. acnes*, *S. aureus*, or *C. albicans* for 7 days at 37 °C under anaerobic conditions in appropriate media with or without shikonin, 1.5 mL samples of cell cultures were harvested by centrifugation at 8000× *g* for 5 min. The assay was repeated four times using four independent cultures.

### 4.7. Cell Agglutination as Determined by EPS Production

To quantify EPS (extracellular polymeric substance) production, we used a previously described method [50]. In brief, *C. acnes*, *S. aureus*, or *C. albicans* were treated with shikonin (0, 1, 2, or 5 μg/mL) at 37 °C for 7 days under anaerobic conditions and then adjusted to ~0.5 at OD_600_ for the assay. Four independent samples were analyzed.

### 4.8. Porphyrin Production Assay

To measure porphyrin production, *C. acnes*, *S. aureus*, or *C. albicans* cells were cultured in RCM, LB, or PDB, respectively, with shikonin (0, 1, 2, or 5 μg/mL) at 37 °C for 7 days under anaerobic condition in the dark. After incubation, 0.5 mL samples of bacterial cultures were mixed with 500 µL of ethyl acetate/acetic acid (4:1) for 10 s by vortexing and centrifuged for 5 min at 10,000× *g*. Upper phases were transferred to new tubes, mixed with 500 µL of 1.5 M HCl for 10 s, and centrifuged for 2 min at 10,000× *g*. Porphyrin production was determined by measuring the absorbances of 200 µL aliquots of lower phases at 405 nm [51]. Four independent samples were analyzed.

### 4.9. Lipase Production Assay

To investigate the effects of shikonin on extracellular lipase production by *C. acnes*, *S. aureus*, and *C. albicans*, cells were inoculated into RCM, LB, or PDB, respectively and incubated for 7 days at 37 °C with shikonin (0, 1, 2, or 5 μg/mL) under anaerobic conditions. Supernatants were then collected by centrifugation at 8000× *g* for 10 min, and 0.1 mL aliquots were mixed with 0.9 mL of substrate buffer [10% (*v*/*v*) of buffer A (3 mg/mL of p-nitrophenyl palmitate in isopropyl alcohol) and 90% (*v*/*v*) of buffer B (1 mg/mL of gum arabic and 2 mg/mL of sodium deoxycholate in 50 mM Na_2_PO_4_ buffer (pH 8.0))] and heated at 40 °C in the dark for 30 min. Lipase reactions were halted by adding 100 µL of 1 M Na_2_CO_3_, followed by centrifugation at 10,000× *g* for 10 min. Supernatant absorbances were measured at 405 nm (OD_405_) [52].

### 4.10. Quantitative Real-Time PCR (qRT-PCR)

*C. acnes* (15 mL) at an initial turbidity of 0.05 at OD_600_ (23 ± 3 × 10^5^ CFU/mL) was inoculated into RCM broth in 15 mL conical tubes and incubated for 3 days at 37 °C without agitation under anaerobic conditions. Cells were then incubated with or without shikonin (5 μg/mL) for an additional 24 h at 37 °C without agitation. RNAlater (RNase inhibitor, Ambion, TX, USA) was used to prevent RNA degradation before cells were harvested. Total RNA was extracted and cleaned using a Qiagen RNeasy mini Kit (Valencia, CA, USA). 

qRT-PCR was used to determine the expression levels of biofilm- and virulence-related genes, namely PPA0721, PPA1796, PPA2105, PPA380, *hly*, PPA0149, *btuR*, *cbiL*, *roxP*, *tly*, and PPA0349, in *C. acnes*. The specific primers and the housekeeping gene (*16s rRNA*) used for qRT-PCR are listed in Table S1. The expression of *16s rRNA* was not affected by shikonin. The qRT-PCR was performed using SYBR Green master mix (Applied Biosystems, Foster City, CA, USA) and an ABI StepOne Real-Time PCR System (Applied Biosystems). At least two independent cultures were used.

qRT-PCR of *S. aureus* was performed after harvesting cells from 10 mL of *S. aureus* cells. Briefly, *S. aureus* at an initial turbidity of 0.05 at OD_600_ (~10^8^ CFU/mL) was inoculated into LB broth in 25 mL flasks and incubated for 3 h at 37 °C with shaking (250 rpm) under aerobic conditions. Cells were then incubated for an additional 3 h at 37 °C and 250 rpm under aerobic conditions, with or without shikonin (10 μg/mL). RNAlater was employed to safeguard against RNA degradation before cell harvest. Total RNA was extracted, cleaned using a Qiagen RNeasy mini Kit, and used to determine the expression levels of biofilm-related genes (*agrA*, *aur*, *hla*, *icaA*, *nuc1*, *RNAIII*, *saeR*, *sarA*, *seb*, and *sigB*). The specific primers used for qRT-PCR are listed in Table S2. As a stable reference gene, 16s rRNA was employed, showing no variation in expression due to shikonin. SYBR Green master mix and an ABI StepOne Real-Time PCR System were utilized for the qRT-PCR procedure, as previously reported [35,41]. At least two independent cultures were used. Gene expression levels were quantified using “Applied Biosystem^TM^ StepOne^TM^ Real-Time PCR System Analysis Software” and normalized versus housekeeping gene expression levels. The analysis was performed using at least two independent cultures and four PCR reactions.

### 4.11. Biofilm Inhibition Analysis on Porcine Skin

This assay used was as previously described [49]. Briefly, fresh quick-frozen porcine skin was obtained from the Korean Federation of Livestock Cooperatives (Seoul, Republic of Korea) and stored at −80 °C until required. Skin pieces (0.5 × 0.5 cm) were sterilized before use by sequential immersion in 70% ethanol and 10% bleach solution for 30 min each. Skin pieces were then washed with sterile water 5 times. *C. acnes* single culture or *C. acnes*, *S. aureus*, and *C. albicans* mixed cultures with shikonin (0, 1, or 5, 10 μg/mL) were then added to the wells of a 12-well plate containing skin pieces and incubated for 7 days at 37 °C without agitation under anaerobic conditions. SEM was used to observe biofilm formation on skin pieces, as described above.

### 4.12. Antimicrobial and Wound-Healing Evaluations in a Murine Model

The animal study was conducted following the experimental protocol approved by the Institute Animal Care and Use Committee at the Korea Research Institute of Bioscience and Biotechnology (No. 200320). Six-week-old C57BL/6J female SPF mice weighing about 20 g were purchased from Daehan Biolink (Kiheung, Republic of Korea). The mice were housed in a temperature- and humidity-controlled room (20.5–23.2 °C and 36.2–56.3% RH). Animals were individually housed in stainless mesh cages and allowed access to rodent chow and filtered water ad libitum. Dorsal hair was removed using a hair cutter and shaving cream under anesthesia by 2,2,2-tribromoethanol (Avertin^®^, Sigma-Aldrich, St. Louis, MO, USA) at 150 mg/kg. Skin was sterilized before incision by applying consecutively povidone iodine and 70% ethanol. Two circular wounds (6 mm) were symmetrically produced on the left and right sides of the dorsum of each mouse using a skin biopsy punch (Kai Medical, Seki, Japan), and the wounds were then burned by applying near-infrared irradiation for 2 min [45]. Twenty-one mice were randomly divided into four groups, as follows: 1, a non-treated and non-infected control group (the sham group); 2, a non-infected shikonin-treated (100 μg/mL) group (the drug-alone group); 3, an infected alone group (the control group); 4, an infected and shikonin-treated (10 μg/mL) group (the shikonin 10 μg/mL group); 5, an infected and shikonin-treated (100 μg/mL) group (the shikonin 100 μg/mL group). Skin incisions in the sham and drug-alone groups were treated with PBS instead. Thirty minutes after infecting right burnt wounds with polymicrobial *S. aureus* (3 × 10^6^ CFU/mL) and *C. albicans* (3 × 10^5^ CFU/mL), 30 μL of PBS containing 10 μL of shikonin was dropped into wounds and incubated for additional 30 min to absorb. Wounds were covered using wound dressing (Tegaderm film; 3M Health Care, St. Paul, MN, USA). Lesion areas were measured at 0, 1, 3, 6, 9, and 12 days post-infection (DPIs), and lesion area percentages versus baseline areas were calculated. 

A cotton stick was used to swab infected skin at DPI 3 to assess bacterial counts, placed in a tube containing 1 mL of PBS, and vigorously vortexed. Serial ten-fold diluted swab solutions (50 μL) were then plated and cultured on TSA plates for *S. aureus* or PDA plates for *C. albicans* for 18 h. Antimicrobial activities were then assessed by counting numbers of bacterial colonies. In addition, skin tissues were harvested at DPI 12 and homogenized in PBS to produce 10% (*w*/*v*) homogenates. Serial ten-fold diluted homogenates were then plated on TSA and PDA, and colony numbers were counted.

For the histopathological study, the spleen and harvested lesioned skin tissues were fixed in 4% formaldehyde and paraffin-embedded. Paraffin blocks were sectioned at 4 μm and stained with H&E (hematoxylin and eosin). Skin re-epithelialization percentages were calculated by measuring the distance covered by regenerated epithelial layers and dividing that by the lesion diameters (6 mm) on DPI 0. Spleen white pulp percentages were calculated by measuring the areas of defined follicle regions in two tissue sections and dividing that by the areas of whole tissue sections.

### 4.13. Shikonin Toxicity Assays Using Nematode and Seed Germination Models

*C. elegans* strain *fer-15(b26)*; *fem-1(hc17)* was used to investigate the toxicity of shikonin in an animal model, as previously described [49]. Briefly, synchronized adult nematodes were washed twice with M9 buffer (3 g/L KH_2_PO_4_, 6 g/L Na_2_HPO_4_, g/L NaCl, 1 mM MgSO_4_) before experiments were started. Approximately 30 nematodes were added to each well of 96-well plates containing M9 buffer (200 µL) with shikonin (0, 1, 2, 5, 10, or 20 μg/mL). Plates were then incubated for 10 days at 25 °C without agitation. This process was repeated independently three times with triplicate samples. Results are presented as percentages of surviving nematodes, assessed based on their reactions to a 30 s exposure to LED light. 

To analyze the effect of shikonin on the germination and growth of Chinese cabbage (*Brassica rapa*), seeds of *B. rapa* were soaked in sterile distilled water for 16 h and rinsed with water three times [53]. Seeds were then placed (10 seeds/plate) on soft agar in Murashige and Skoog plates (0.7% agar and 0.86 g/L Murashige and Skoog) and incubated at room temperature (24 °C) for 10 days. Seed germination percentages and total plant lengths were measured. Four independent experiments were performed at each concentration.

### 4.14. Statistical Analysis

The number of replications for the assays is indicated above, and the findings are presented as means ± standard deviations. Statistical analysis was conducted utilizing one-way ANOVA followed by Dunnett’s test in SPSS version 23 (SPSS Inc., Chicago, IL USA). Statistical significance was determined at *p* values of <0.05, with asterisks denoting significant disparities between treated and untreated samples.

## Data Availability

The datasets used and/or analyzed during the current study are available from the corresponding author on reasonable request. Data are contained within the article.

## Supplementary Materials

The following supporting information can be downloaded at https://www.mdpi.com/article/10.3390/ijms25042426/s1.
