# Supplementary material for: Inhibition of Biofilm Formation in Cutibacterium acnes, Staphylococcus aureus, and Candida albicans by the Phytopigment Shikonin
Source: Int J Mol Sci. 2024 Feb 19;25(4):2426. doi: 10.3390/ijms25042426 (PMC10888572; doi:10.3390/ijms25042426)
Supplement: Supplementary file 1 [file ijms-25-02426-s001.zip › ijms-2866971-supplementary.pdf]

Table S1. qRT-PCR primer sequences used for *C. acnes*.

Gene	Description	Primer
PPA0721	Invasion-associated protein p60	Forward 5'-AGA AGA AGG AAC AGG AGG CC-3'<br>Reverse 5'-TCA GGA ATC GGC ACT TGA CT-3'
PPA1796	Lipase	Forward 5'-CTC AAG GTT CGT GAA CGA GT-3'<br>Reverse 5'-GCC ATA GAG CTC CTT GTT GA-3'
PPA2105	Lipase	Forward 5'-GAT TTC CTT AGC ACG TGG AG-3'<br>Reverse 5'-GAT GAC GGT GTA GGC GAT AC-3'
PPA380	Hyaluronate lyase	Forward 5’-CGC TCT GAA GGA TTC GTC-3’<br>Reverse 5’-GTC GTG CAG GAT ACA CAT GA-3’
hly	Hyaluronate lyase	Forward 5’-CAA CAT CGC CGT GTT TAT TG-3’<br>Reverse 5’-CCC ATG ACG ACG TAG AGG AT-3’
PPA0149	Glycosyltransferase	Forward 5'-AGT ACA TGG CTT CCC GAG TG-3'<br>Reverse 5'-CTT GGG ACT CGA AGT TGA GC-3'
btuR	Cobinamide/cobalamin adenosyltransferase	Forward 5'-GGA AGA TGC TCT TCG GGC GCT-3'<br>Reverse 5'-GCC TCA GGG TTC TCC GCA GC-3'
cbiL	Precorrin-2 C(20)-methyltransferase	Forward 5'-GCG CGA GGC AGA CGT GAT CC-3'<br>Reverse 5'-GAC ACC GGA CCT CTC CCG CA-3'
roxP	Radical oxygenase	Forward 5'-GCA TCT AGC CCT CTC ACC AT-3'<br>Reverse 5'-CTG AGA GTC CGG TAG GTG GT-3'
tly	Lysis of red blood cells	Forward 5'-CAG GAC GTG ATG GCA ATG CGA-3'<br>Reverse 5'-TCG TTC ACA AGA CCA CAG TAG C-3'
PPA0349	Polysaccharide capsule biosynthesis	Forward 5’-CTT CTT CGT CGA CCA GTT CC-3'<br>Reverse 5’-TCA GCT GTC TCG TCA ACA CC-3'
16s rRNA	16S ribosomal RNA (Housekeeping gene)	Forward 5'-GGG GCT TAA CCC TGA GCG TGC-3'<br>Reverse 5'-TTC GCT CCC CAC GCT TTC GC-3'

Table S2. qRT-PCR primer sequences used for *S. aureus*.

Gene	Name	Primer
agrA	Quorum-sensing regulator A	Forward 5'-TGA TAA TCC TTA TGA GGT GCT T-3'<br>Reverse 5'-CAC TGT GAC TCG TAA CGA AAA-3'
aur	Zinc metalloprotein ase aureolysin	Forward 5'-ACC GTG TGT TAA TTC GTG TGC TA-3'<br>Reverse 5'-ATG GTC GCA CAT TCA CAA GTT T-3'
hla	α-Hemolysin	Forward 5'-CGG CAC ATT TGC ACC AAT AAG GC-3'<br>Reverse 5'-GGT TTA GCC TGG CCT TCA GC-3'
icaA	Intercellular adhesion A	Forward 5'-TGA ACC GCT TGC CAT GTG-3'<br>Reverse 5'-CAC GCG TTG CTT CCA AAG A-3'
nuc1	Nuclease	Forward 5'-CAC CTG AAA CAA AGC ATC CTA A-3'<br>Reverse 5'-TAT ACG CTA AGC CAC GTC CAT-3'
RNAIII	Transcriptiona l regulator	Forward 5'-ATC GAC ACA GTG AAC AAA TTC AC-3'<br>Forward 5'-CTC TAC TAG CAA ATG TTA CTC AC-3'
saeR	Response regulator	Forward 5'-GCC TTA ACT TTA GGT GCA GAT GAC TAT GTC-3'<br>Forward 5'-CGA CAG TTG TTC AAC TGG TTG ATG ATG G-3'
sarA	Transcriptiona l regulator	Forward 5'-GAG TTG TTA TCA ATG GTC-3'<br>Reverse 5'-GTT TGC TTC AGT GAT TCG-3'
seb	Enterotoxin B	Forward 5'-TGT TCG GGT ATT TGA AGA TGG -3'<br>Reverse 5'-CGT TTC ATA AGG CGA GTT GTT-3'
sigB	RNA Polymerase sigma factor	Forward 5'-AAG TGA TTC GTA AGG ACG TCT-3'<br>Reverse 5'-TCG ATA ACT ATA ACC AAA GCC T-3'
spa	Protein A	Forward 5'-ACC AGA AAC TGG TGA AGA AAA TCC-3'<br>Reverse 5'-TAA CGC TGC ACC TAA GGC TAA TG-3'
16s rRNA	A component of ribosomes	Forward 5'-TGT TTG ACG ATG TTT GAG CA-3'<br>Reverse 5'-CCT TCC TCC AGT TCA GAT GC -3'

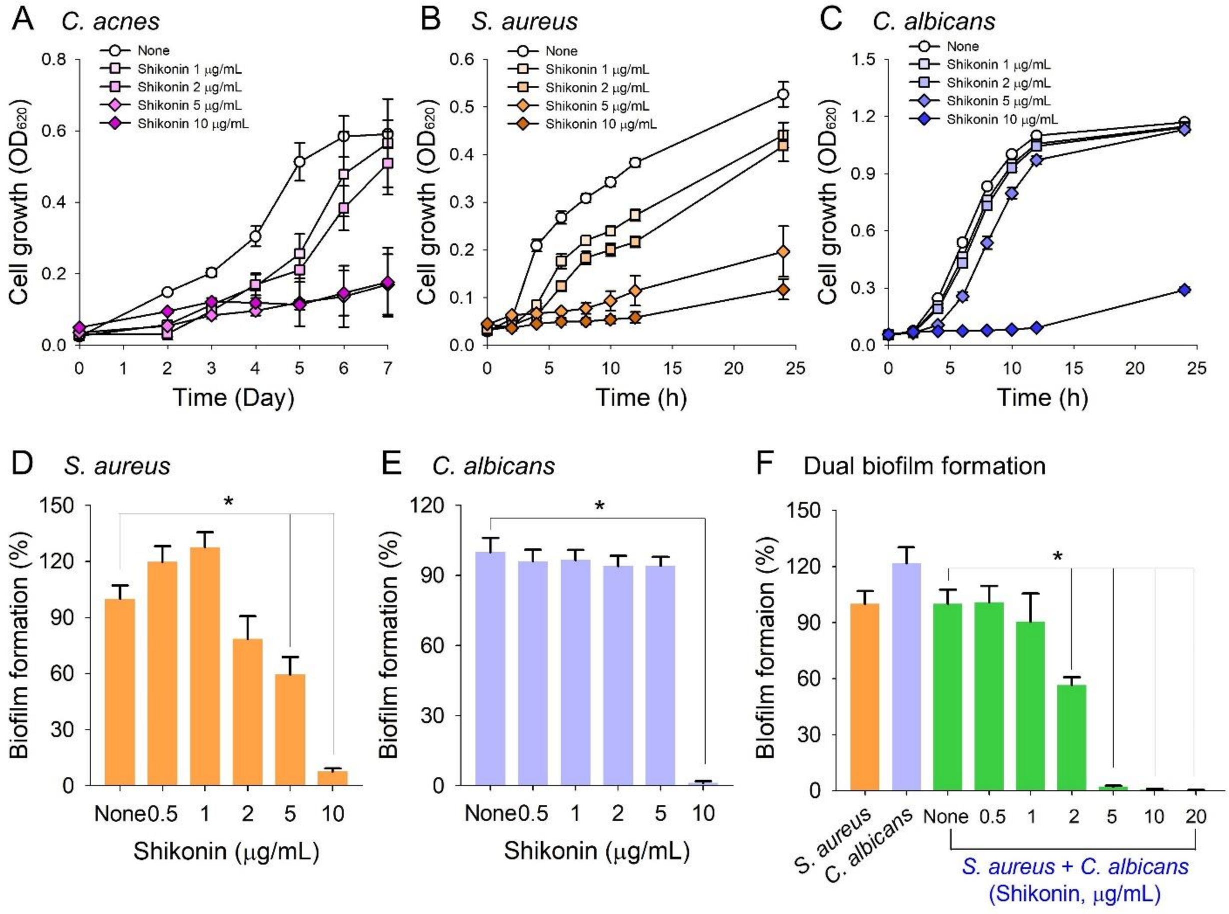

Figure S1. Planktonic cell growths of (A) *C. acnes*, (B) *S. aureus*, and (C) *C. albicans* in the presence of shikonin under anaerobic conditions in 96-well plates. Biofilm formations by the (D, E) two microbes separately and (F) mixed microbes after culture for 1 day under aerobic conditions.

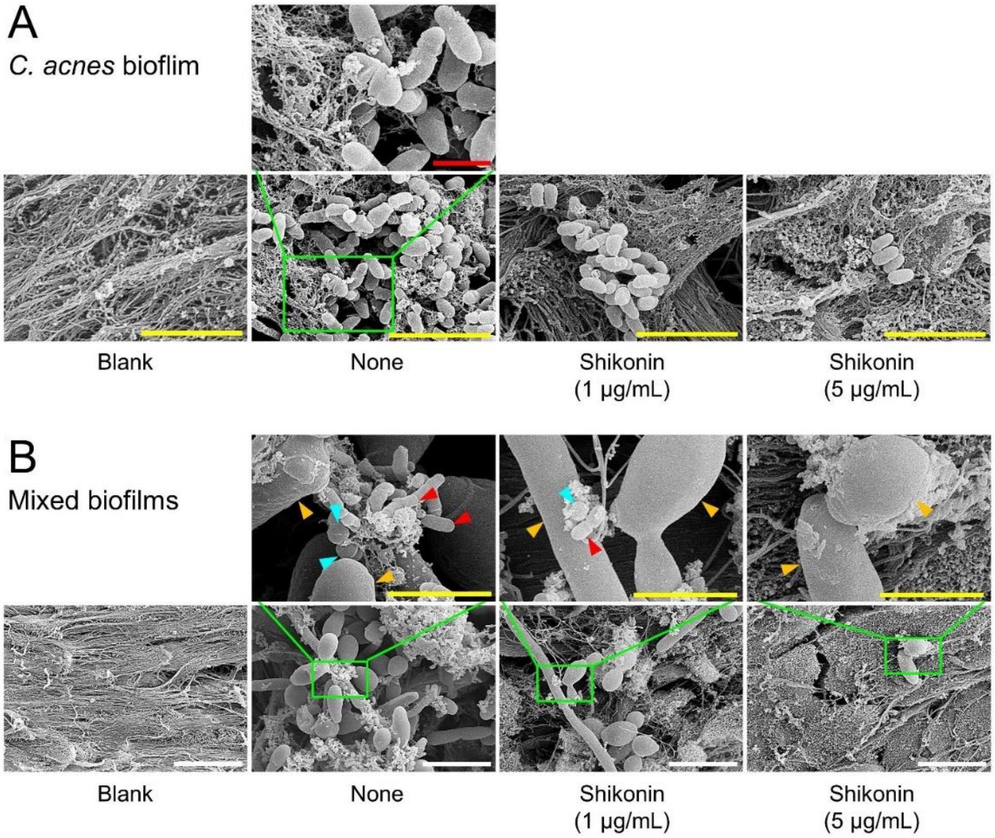

Figure S2. Preventation of biofilm formation on porcine skin. Inhibitory effects of shikonin on biofilm development by (A) *C. acnes* and (B) *C. acnes/S. aureus/C. albicans* on porcine skin. SEM was used to observe biofilms after 7 days of culture under anaerobic conditions. Yellow, red, and white scale bars indicate 4, 1, and 10 µm, respectively. Red, cyan, and orange arrowheads indicate *C. acnes*, *S. aureus*, and *C. albicans* cells, respectively.

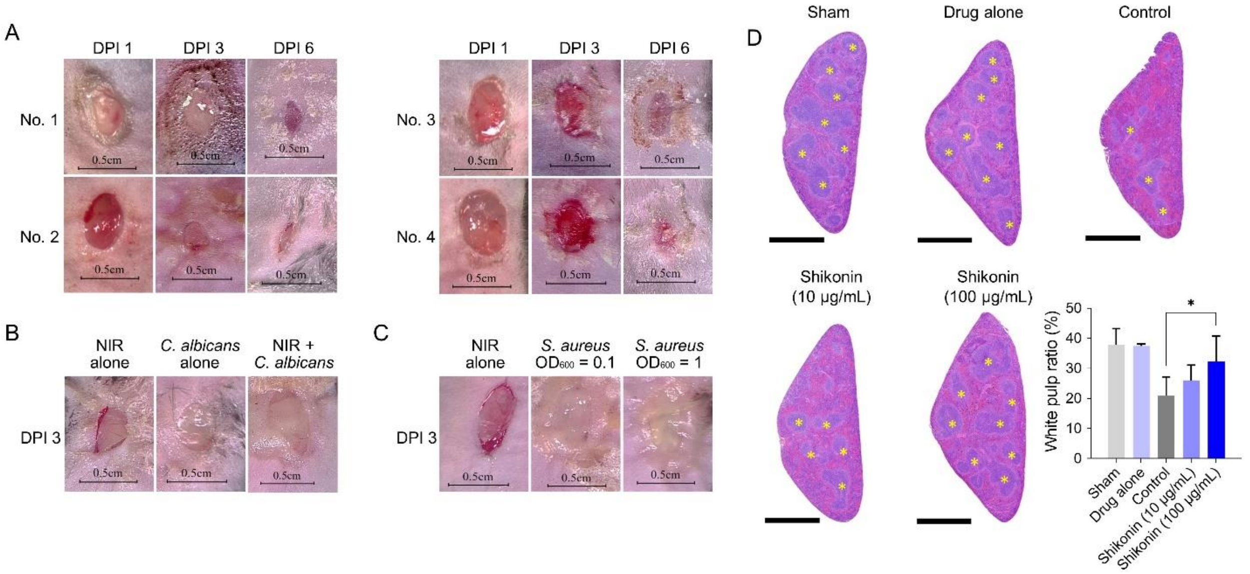

Figure S3. (A) Images of lesions induced by *C. albicans* or *S. aureus* with or without burning. *C. albicans* infection without burning did not result in inflammatory lesion progression at DPI 6. NIR application aggravated skin inflammation induced by (B) *C. albicans* of OD_{600} = 1 (3×10^5 CFU) and (C) *S. aureus* of OD_{600} = 0.1 (3×10^6 CFU) and 1 (3×10^7 CFU). (D) Spleen histopathology after infecting wounds with *S. aureus* and *C. albicans* after DPI 12. Percentage white pulp areas (asterisk) are shown in the bar chart. Scale bars represent 1 mm. Means ± standard deviations of at least 3 animals are presented. *, $p < 0.05$ *vs.* infected non-treated controls.
